# Renal Artery Denervation Combined with Pulmonary Vein Isolation in Patients with Heart Failure and Atrial Fibrillation: Pilot Study: Renal Artery Denervation in Treatment of Atrial Fibrillation and Heart Failure

**DOI:** 10.3390/jcm14051727

**Published:** 2025-03-04

**Authors:** Tomasz Skowerski, Mariusz Skowerski, Iwona Wozniak-Skowerska, Andrzej Hoffmann, Andrzej Kułach, Andrzej Ochała, Katarzyna Mizia-Stec, Zbigniew Gasior, Grzegorz Smolka

**Affiliations:** 1 Department of Cardiology, School of Health Sciences, Medical University of Silesia, 45/47 Ziolowa Street, 40-635 Katowice, Poland; mskowerski@sum.edu.pl (M.S.); andrzejkulach@gmail.com (A.K.);; 2 1st Department of Cardiology, School of Medicine in Katowice, Medical University of Silesia, 40-635 Katowice, Poland; iskowerska@hoga.pl (I.W.-S.);; 3 Department of Cardiology and Structural Heart Diseases, Medical University of Silesia, 40-635 Katowice, Poland

**Keywords:** renal denervation, atrial fibrillation, pulmonary vein isolation

## Abstract

**Background:** Heart failure (HF) is a progressive condition associated with reduced life expectancy and quality of life. Atrial fibrillation (AF), the most common arrhythmia in HF patients, significantly worsens symptoms and outcomes. The coexistence of HF and AF is linked to higher morbidity and mortality rates, with a bidirectional relationship exacerbating both conditions. The recent evidence has suggested that combining pulmonary vein isolation (PVI) with renal denervation (RDN) may offer a promising strategy for reducing AF burden and enhancing patient outcomes. **Methods:** This prospective interventional clinical trial aimed to assess the safety and effectiveness of a combined RDN and PVI approach compared to PVI alone. Eighteen patients, aged 18 to 80 years, with paroxysmal or persistent AF and HF (left ventricular ejection fraction [LVEF] < 50%) were enrolled. RDN was performed under general anesthesia using the four-electrode Symplicity Spyral catheter and Symplicity G3 radiofrequency generator (Medtronic). Patients were randomized to the RDN+PVI group (*n* = 7) or the PVI-only group (*n* = 11). The groups were similar in age (59 ± 8.4 years vs. 62.5 ± 11.08 years, *p* = NS) and baseline characteristics, including hypertension, obesity, and impaired left ventricular function (LVEF 35.86% vs. 38.54%, RDN+PVI vs. PVI only; *p* = NS). **Results:** Over a mean follow-up of 24 months, one patient died, ten were hospitalized, six underwent repeat PVI, and eight achieved AF freedom. Patients in the RDN+PVI group were significantly more likely to remain AF-free (n = 6 vs. 2; *p* = 0.0063). The need for repeat ablation was higher in the PVI-only group (54.5% vs. 0%), though this did not reach statistical significance. Hospitalization rates and changes in ejection fraction were similar between groups. Importantly, no procedural complications were observed. **Conclusions:** Combining RDN with PVI is a safe hybrid approach for AF management in HF patients, showing promising efficacy in reducing AF recurrence. Larger randomized studies are needed to confirm these findings and further explore this novel therapeutic strategy.

## 1. Background

The global prevalence of heart failure (HF) is substantial, affecting approximately 1–2% of the adult population. By 2030, the burden of HF is expected to increase by over 25% due to aging populations and rising risk factors such as hypertension and diabetes. HF is a progressive disease that leads to serious complications and significantly reduces life expectancy.

The 5-year survival rate for HF patients is less than 50%, depending on the type of HF. Patients with NYHA Class IV have an annual mortality rate that can exceed 50%. Additionally, quality of life is severely impaired, with patients experiencing significant limitations in daily activities, depression, and a reduced ability to function, which further affects prognosis [1,2,3,4].

Atrial fibrillation (AF) significantly impairs the survival rates of heart failure (HF) patients. The coexistence of AF and HF is associated with a substantial increase in mortality and morbidity. Several studies have consistently shown that patients with both conditions have poorer outcomes compared to those with HF alone. The prevalence of AF in HF patients is estimated at 30–50%. There is a bidirectional relationship between heart failure and AF [1,5].

Pulmonary vein isolation (PVI) is increasingly recognized as the preferred treatment strategy for atrial fibrillation (AF) in patients with heart failure (HF), particularly those with heart failure with reduced ejection fraction (HFrEF). As outlined in the 2024 European Society of Cardiology (ESC) Guidelines for the management of atrial fibrillation, PVI plays a critical role in restoring and maintaining sinus rhythm. PVI is associated with significant improvements in left ventricular function, reductions in heart failure-related hospitalizations, and enhanced survival outcomes in this patient population [6].

PVI is highly effective for managing atrial fibrillation (AF), with sinus rhythm maintained in 60–80% of patients after a single procedure. It leads to significant symptom relief, improved quality of life, and reduced AF recurrence, but the outcomes are worse in coexisting HF [4,6,7,8,9].

Renal denervation (RDN) was initially developed as a treatment for resistant hypertension by targeting the sympathetic nervous system. Recent evidence, however, suggests that combining pulmonary vein isolation (PVI) with RDN may provide additional benefits in reducing the burden of atrial fibrillation (AF), lowering hospitalization rates, and improving mortality and quality of life in patients with AF. Studies exploring the synergistic effects of PVI and RDN have indicated that RDN may contribute to more effective rhythm control by modulating autonomic influence on the heart, which plays a role in AF pathogenesis. This is the first study assessing the feasibility, efficacy, and safety of RDN in patients with both HF and AF [10,11,12,13,14,15,16].

## 2. Methods

This study was a prospective interventional clinical trial designed to evaluate the safety and efficacy of RDN with PVI in comparison to PVI only in patients with AF and HF. This study was approved by the Ethics Committee (Medical University of Silesia Ethical Committee, Approval Code: KNW/022/KB1/67/18, Approval Date: 28 May 2018). and conformed to the Declaration of Helsinki. This study was conducted at the Medical University of Silesia as a statutory project of the Department of Cardiology, School of Medical Sciences. The trial was officially registered within the university in 2018 under the registration number PCN-1-218/N/9/K. As this was a single-center study, it was not registered in ClinicalTrials.gov or any other international registry. Informed written consent was obtained from every patient enrolled in this study. Patients were enrolled in two cardiology clinics in Poland. RDN procedures were performed by two experienced operators certified by the RDN electrode manufacturer (Medtronic Minneapolis, MN, USA).

PVI procedures were carried out by a team of three electrophysiologists with more than 15 years of clinical practice.

### 2.1. Participants

The study population included patients aged 18 to 80 years with paroxysmal or persistent atrial fibrillation and heart failure (defined as left ventricular ejection fraction < 50%). At least one episode of AF had to be documented by ECG within 1 year before enrollment for patients with paroxysmal AF. Subjects with persistent AF were defined as having a continuous AF episode lasting longer than 7 days but less than 1 year.

Patients with chronic kidney disease (eGFR ≤ 30 mL/min/1.73 m^2^ assessed using the Cockcroft–Gault formula), autoimmune disorders, previous RDN procedures, PCI or CABG within 6 months prior to enrollment, stroke within 6 months prior to enrollment, severe stenosis and/or regurgitation of any heart valve, LVEF > 50%, lower extremity arterial disease, cancer, or pregnancy were excluded from this study. The eligibility criteria are presented in Table 1. Renal hypertension was excluded through clinical assessment, including imaging studies (renal Doppler ultrasound or CT angiography) and laboratory evaluations. Renal arteriography was performed prior to renal denervation to exclude stenosis of renal artery.

On admission, a full baseline evaluation was performed, including medical history, physical examination, medication review, 12-lead ECG, and transthoracic and transesophageal echocardiogram.

### 2.2. RDN Procedure

Renal denervation was performed under general anesthesia via the femoral artery using a 4F dedicated ablation catheter—the four-electrode Symplicity Spyral catheter (Medtronic; Galway, Ireland) and the Symplicity G3 radio frequency generator (Medtronic; Minneapolis, MN, USA). Every patient received heparin in doses adequate to achieve an activated clotting time (ACT) of >300 s. The radiofrequency ablations were performed in a spiral pattern in main renal arteries, branch vessels, and additional renal arteries between 3 and 8 mm in diameter. Femoral artery closure devices (Angio-Seal; Terumo Interventional Systems, Tokyo, Japan) were used in every patient.

### 2.3. PVI Procedure

At the beginning of each PVI procedure, rotational angiography of the left atrium was performed. After analyzing the obtained scans, the operator chose either cryoablation or radiofrequency ablation.

If cryoablation was chosen, PVI was executed using a cryoballoon catheter (Arctic Front Advance Pro; Medtronic Inc., Minneapolis, MN, USA) with 1–2 freezes/vein, each lasting 180–240 s.

In the case of RF ablation, a CARTO or EnSite system was utilized to create a map of the left atrium and ablation was performed using a SmartTouch electrode (Biosense Webster, Irvine, CA, USA) or TactiCath (Abbott, Plymouth, MN, USA).

The success of the PVI procedure was confirmed by a multielectrode mapping catheter within each PV, demonstrating the disappearance or dissociation of all PV potentials and an exit block.

### 2.4. Study Design

The first procedure performed after enrollment was PVI. The efficacy of the PVI procedure was assessed after a 6-month blinding period. Then, patients were randomized into the RDN group or standard-of-care/pharmacology group. Patients were randomized using a computer-generated random sequence, ensuring unbiased allocation to either the RDN+PVI group or the PVI-only group. Additionally, allocation concealment was ensured using sealed, opaque envelopes opened only after patient enrollment.

The study endpoints were atrial fibrillation episodes, hospitalization (due to AF recurrence), reablation (due to AF recurrence), and death.

Patient enrollment and procedures were conducted from 2019 to 2021, coinciding with the COVID-19 pandemic. The pandemic disrupted healthcare services in Poland, reducing patient availability and procedure frequency, which contributed to the limited sample size.

### 2.5. Statistical Analysis

Statistical analyses were conducted using SPSS version 25.0 (IBM Corp., Armonk, NY, USA) and MedCalc version 14.8.1 (MedCalc Software, Ostend, Belgium). Continuous variables were reported as mean ± standard deviation (SD) or median with interquartile range (IQR), depending on their distribution. Categorical variables were presented as absolute numbers and percentages. The Shapiro–Wilk test was employed to evaluate the distribution of continuous variables. Since all continuous variables exhibited non-normal distributions, nonparametric tests, including the two-tailed Mann–Whitney U test or Kruskal–Wallis test, were applied. The chi-square test was used to assess the significance of proportions within contingency tables. For logistic regression analysis, variables with a *p*-value < 0.1 in the univariate analysis were included to identify independent predictors of outcomes. Statistical significance was consistently defined as a *p*-value < 0.05 throughout all analyses. A post hoc power analysis was conducted to assess the adequacy of the sample size for detecting differences in AF freedom rates between the RDN+PVI and PVI-only groups. Based on the observed proportions (85.7% vs. 18.2%) and a significance level of 0.05, the calculated effect size (Cohen’s h) was 1.253. This study achieved a power of 80.3%, indicating sufficient sensitivity to detect a statistically significant difference between the groups.

## 3. Results

The mean follow-up time was 24 months. A total of 18 patients were enrolled into this study, then were randomized to receive either renal denervation and pulmonary vein isolation (7 pts) or pulmonary vein isolation only (11 pts). The baseline characteristics of study groups is presented in Table 2.

The mean age was 59 (±8.4) years in the RDN+PVI group, and 62.5 (±11.08) years in the PVI group (*p* = NS); the whole study group comprised mostly male subjects. The patients suffered from arterial hypertension (100% of pts in both groups), were obese (mean BMI 28.3, 28.7; RDN+PVI, PVI only), had heart failure (mainly in NYHA II class), and impaired left ventricular function (LVEF 35.86 vs. 38.54; RDN+PVI vs. PVI only; *p* = NS).

The HF and AF were treated according to the guidelines, all of the subjects received beta-blocker, anticoagulation (mostly NOACs), the majority of them received diuretics, spironolactone, ACEI, or ARNI. The ICD/CRT was implanted in 3/7 pts in the RDN+PVI group and 2/11 pts in the PVI-only group.

Medication adherence was monitored during follow-up and was found to be consistent across groups, with no significant differences observed (*p* = 0.56). Additionally, changes in renal function (e.g., estimated glomerular filtration rate) were assessed pre- and post-procedure. No significant declines in renal function were observed in either group.

A subgroup analysis comparing patients undergoing cryoablation versus RF ablation was conducted to address variability introduced by operator choice. The analysis revealed no significant differences in AF freedom rates between the two techniques (*p* = 0.45). This suggests that the choice of ablation modality did not impact the primary outcome, although larger studies may be needed to confirm this finding.

The mean follow-up time was 24 months. During the follow-up, one patient died, ten patients were hospitalized (one or more times), six patients underwent PVI again, and eight patients were free of AF. There were no complications.

Patients from RDN+PVI group were significantly more often free from AF (6 vs. 2 pts; RDN+VI vs. PVI only; *p* = 0.0063). The rate and number of hospitalizations per patient did not differ between study groups. Although the rate of reablation was 54.5% in the PVI group vs. 0% in the RDN+PVI group, the difference did not reach the statistical significance.

The ejection fraction did not differ significantly in both groups—comparing data before PVI or PVI+RDN and after procedures. The complete follow-up data are presented in Table 3.

## 4. Discussion

### 4.1. Main Findings

This study demonstrates that combining renal sympathetic denervation (RDN) with pulmonary vein isolation (PVI) improves rhythm control in patients with atrial fibrillation (AF) and heart failure (HF), particularly those with concomitant hypertension. In our cohort, the RDN+PVI group achieved a significantly higher rate of freedom from AF (86% vs. 18%; *p* = 0.0063) compared to PVI alone. Notably, no procedural complications were observed, reinforcing the safety profile of RDN when performed in conjunction with PVI. These findings highlight the potential benefit of this hybrid approach, which targets both the triggers (PVI) and autonomic substrate (RDN) of AF. The lack of significant changes in renal function following the procedure suggests that RDN is a safe intervention in this regard. However, confounding variables such as lifestyle behaviors (e.g., smoking and dietary habits) were not collected and represent a limitation of this study.

The autonomic nervous system plays a central role in AF pathogenesis, with heightened sympathetic activity contributing to atrial ectopy, nerve sprouting, and structural remodeling. Renal sympathetic denervation interrupts sympathetic outflow from renal afferent nerves, reducing systemic norepinephrine levels, atrial fibrosis, and electrical heterogeneity [17,18]. Experimental studies by Yamada et al. demonstrated that RDN reverses atrial structural remodeling, particularly in heart failure models, where autonomic dysregulation accelerates AF progression [19].

Furthermore, Qiu et al. observed that RDN significantly improved ventricular rate control in patients with persistent AF and hypertension, independent of blood pressure reductions, by modulating AV node function [13]. These findings suggest that the benefits of RDN extend beyond hypertension control to directly impacting AF mechanisms, including nerve activity and arrhythmic substrate modulation. Nammas et al. confirmed that these effects persist even in hypertensive patients with chronic kidney disease, further emphasizing the role of autonomic modulation in reducing AF burden [14].

Recent studies have highlighted the role of kidney-acting drugs, such as sodium-glucose cotransporter-2 (SGLT2) inhibitors and mineralocorticoid receptor antagonists, in the management of AF and heart failure. These drugs modulate neurohormonal pathways and may reduce atrial remodeling and fibrosis. For example, SGLT2 inhibitors have been shown to lower the risk of incidence of AF in patients with type 2 diabetes [20]. Incorporating these agents into future treatment strategies may provide additive benefits alongside interventions such as RDN. We must highlight the fact that this study was conducted before registration of SGLT2 for HF in 2020 in Poland.

### 4.2. Safety of the Hybrid Approach in Heart Failure

This study is the first to evaluate the safety and efficacy of the RDN+PVI hybrid approach specifically in patients with heart failure. Heart failure patients represent a high-risk group for procedural complications due to advanced comorbidities and reduced functional reserve. Despite these concerns, our study demonstrated that the RDN+PVI procedure was not associated with any significant complications. This aligns with findings from RDN trials, such as SPYRAL HTN, and RADIOSOUND-HTN, which have consistently reported a favorable safety profile for RDN in hypertensive populations [21].

The absence of vascular complications, acute kidney injury, or arrhythmia-related adverse events in our cohort supports the feasibility of performing RDN alongside PVI in this vulnerable population. Importantly, the synergistic benefits observed in rhythm control suggest that this combined strategy can improve outcomes without compromising safety. Future large-scale studies are required to confirm these findings and establish guidelines for its use in heart failure patients.

### 4.3. Comparison with Existing Studies

The results of our study align with prior evidence supporting the synergy between RDN and PVI. The ERADICATE-AF trial, which randomized 302 patients with paroxysmal AF and hypertension, demonstrated that adding RDN to PVI significantly increased freedom from AF at 12 months (72.1% vs. 56.5%; HR = 0.57; *p* = 0.006) [18]. This finding highlights the additive role of RDN in improving long-term rhythm control. Although the ERADICATE trial provided valuable insights, its use of a non-dedicated system for renal denervation introduces a significant source of bias, potentially impacting the reliability and generalizability of the findings.

A meta-analysis by Ukena et al. reviewed six randomized studies with 689 patients undergoing RDN+PVI or PVI alone. The pooled results showed a 57% reduction in AF recurrence (OR = 0.43; 95% CI: 0.32–0.59) in the RDN+PVI group [22]. Moreover, the addition of RDN was associated with significant reductions in systolic blood pressure and improved estimated glomerular filtration rate (eGFR) [22].

Pokushalov et al. provided early evidence that combining RDN with PVI reduces AF recurrence in hypertensive patients with refractory AF [23] In their cohort, AF recurrence was observed in 29% of the RDN+PVI group compared to 56% of the PVI-only group (*p* < 0.05). The RADIOSOUND-HTN trial further demonstrated that newer RDN techniques, including branch artery ablation, enhance efficacy without increasing procedural complications [24].

### 4.4. Clinical Implications

The combination of RDN and PVI has significant clinical implications, particularly for AF patients with hypertension, heart failure, or chronic kidney disease.

1. Improved Rhythm Control: The significantly higher freedom from AF observed in our study and others suggests that RDN+PVI targets both the triggers and substrate of AF, reducing recurrence rates [1,6,7].
2. Reduced Blood Pressure: While RDN’s primary role is autonomic modulation, it also contributes to blood pressure reductions, which are independently associated with lower AF recurrence [8,9,21].
3. Enhanced Patient Selection: Patients with sympathetic overactivation, such as those with resistant hypertension or HF, may derive the greatest benefit from RDN+PVI [5,6].

Given the favorable safety profile observed in our study and confirmed by trials such as SPYRAL HTN and ERADICATE-AF, integrating RDN into current AF management protocols may offer a comprehensive therapeutic strategy for patients with complex comorbidities [7,10].

## 5. Conclusions

Combining renal sympathetic denervation with pulmonary vein isolation represents a promising and effective strategy for rhythm control in AF patients with heart failure. By targeting both autonomic dysfunction and arrhythmic triggers; this hybrid approach offers superior outcomes, particularly in patients with hypertension or heightened sympathetic activity. Larger trials are warranted to confirm these findings and establish the long-term clinical benefits of RDN+PVI

### Limitations

The small sample size of this pilot study represents a limitation, although the post hoc power analysis indicated that the sample size was sufficient to detect significant differences. Larger, multicenter studies are needed to validate these results in a broader population. While variability in operator preference for ablation modality may introduce heterogeneity, the subgroup analysis showed no significant difference in outcomes between cryoablation and RF ablation. Standardization of procedural approaches should be considered in future studies to ensure consistency across centers.

## Figures and Tables

**Table 1:** Inclusion and exclusion criteria.

Inclusion Criteria
Heart failure with symptoms and left ventricular ejection fraction (LVEF) < 50%. Paroxysmal atrial fibrillation (at least one episode documented with ECG recording) or persistent atrial fibrillation (lasting >7 days but <1 year). Age between 18 and 80 years old.
**Exclusion Criteria**
Chronic kidney disease (eGFR ≤ 30 mL/min/1.73 m^2^, assessed using the Cockcroft–Gault formula). Autoimmune disorders. Previous renal denervation (RDN) procedure. Percutaneous coronary intervention (PCI) or coronary artery bypass grafting (CABG) within 6 months before enrollment. Any stroke within 6 months prior to enrollment. Severe stenosis and/or regurgitation of any heart valve. Preserved left ventricular ejection fraction (LVEF > 50%). Lower-extremity arterial disease. Cancer. Pregnancy.

**Table 2:** Baseline characteristics.

	RDV + PVI n = 7	PVI Only n = 11
Age, SD	59 ± 8.4	62.5 ± 11.08
Male, n, %	6, 86%	11, 100%
BMI, SD	28.3 ± 4.1	28.7 ± 2.9
NYHA class		
I	0	2
II	6	9
III	1	0
Left ventricular ejection fraction	35.86 ± 9.2	38.54 ± 7.6
Left atrial diameter	47.8 ± 6.1	44.2 ± 3.8
Arterial hypertension, n, %	7, 100%	11, 100%
Diabetes, n, %	3, 42.8%	3, 27.2%
Coronary artery disease, n, %	4, 57%	4, 36%
Sinus rhythm at ablation, n, %	4, 57%	4, 36%
eGFR, SD	84.14 ± 8.4	74.45 ± 11.42
Medications:		
NOAC	6/7	9/11
VKA	1/7	2/11
Betablocker	100%	100%
diuretic	6/7	7/11
Spironolactone/eplerenone	7/7	9/11
ARNI	2/7	1/11
ACEI	5/7	8/11
ICD/CRT	3/7	2/11

**Table 3:** Follow-up results.

	RDN+PVI n = 7	PVI Only n = 11	*p*
Absence of AF, n, %	6 (85.7%)	2 (18.2%)	*p* = 0.0063
Hospitalization, n, %	3 (42.85%)	7 (63.6%)	*p* = 0.4
Death, n, %	0	1 (9.1%)	*p* = 0.425
Reablation, n, %	0	6 (54.5%)	*p* = 0.0969

## Data Availability

The raw data supporting the conclusions of this article will be made available by the authors on request.

## References

[1] McDonagh T.A., Metra M., Adamo M., Baumbach A., Böhm M., Burri H., Čelutkiene J., Chioncel O., Cleland J.G.F., Coats A.J.S. (2021). 2021 ESC Guidelines for the Diagnosis and Treatment of Acute and Chronic Heart Failure. Eur. Heart J..

[2] Shahim B., Kapelios C.J., Savarese G., Lund L.H. (2023). Global Public Health Burden of Heart Failure: An Updated Review. Card. Fail. Rev..

[3] Diamond J., Ayodele I., Fonarow G.C., Joynt-Maddox K.E., Yeh R.W., Hammond G., Allen L.A., Greene S.J., Chiswell K., Devore A.D. (2023). Quality of Care and Clinical Outcomes for Patients with Heart Failure at Hospitals Caring for a High Proportion of Black Adults: Get with the Guidelines-Heart Failure Registry. JAMA Cardiol..

[4] Johansson I., Joseph P., Balasubramanian K., McMurray J.J.V., Lund L.H., Ezekowitz J.A., Kamath D., Alhabib K., Bayes-Genis A., Budaj A. (2021). Health-Related Quality of Life and Mortality in Heart Failure The Global Congestive Heart Failure Study of 23000 Patients from 40 Countries. Circulation.

[5] Mulder B.A., Rienstra M., Van Gelder I.C., Blaauw Y. (2022). Update on Management of Atrial Fibrillation in Heart Failure: A Focus on Ablation. Heart.

[6] Van Gelder I.C., Rienstra M., Bunting K.V., Casado-Arroyo R., Caso V., Crijns H.J.G.M., De Potter T.J.R., Dwight J., Guasti L., Hanke T. (2024). 2024 ESC Guidelines for the Management of Atrial Fibrillation Developed in Collaboration with the European Association for Cardio-Thoracic Surgery (EACTS). Eur. Heart J..

[7] Foroutan F., Rayner D.G., Ross H.J., Ehler T., Srivastava A., Shin S., Malik A., Benipal H., Yu C., Alexander Lau T.H. (2023). Global Comparison of Readmission Rates for Patients with Heart Failure. J. Am. Coll. Cardiol..

[8] Shah K.S., Xu H., Matsouaka R.A., Bhatt D.L., Heidenreich P.A., Hernandez A.F., Devore A.D., Yancy C.W., Fonarow G.C. (2017). Heart Failure with Preserved, Borderline, and Reduced Ejection Fraction 5-Year Outcomes. J. Am. Coll. Cardiol..

[9] Siontis K.C., Yao X., Noseworthy P.A. (2018). Atrial Fibrillation in Heart Failure Syndromes: Does It Matter More in Some than in Others?. Eur. Heart J..

[10] Huang B., Yu L., Zhou L., Jiang H. (2015). Renal Denervation for the Treatment of Atrial Fibrillation in Hypertensive Patients or Beyond?. Int. J. Cardiol..

[11] Yi X., Feng G., Jiang X. (2017). A Potential and Lionhearted Soldier for Atrial Fibrillation Accompanied with Heart Failure: Renal Denervation. Int. J. Cardiol..

[12] Nawar K., Mohammad A., Johns E.J., Abdulla M.H. (2022). Renal Denervation for Atrial Fibrillation: A Comprehensive Updated Systematic Review and Meta-Analysis. J. Hum. Hypertens..

[13] Qiu M., Shan Q., Chen C., Geng J., Guo J., Zhou X., Qian W., Tang L., Yin Y. (2016). Renal Sympathetic Denervation Improves Rate Control in Patients with Symptomatic Persistent Atrial Fibrillation and Hypertension. Acta Cardiol..

[14] Nammas W., Airaksinen J.K.E., Paana T., Karjalainen P.P. (2016). Renal Sympathetic Denervation for Treatment of Patients with Atrial Fibrillation: Reappraisal of the Available Evidence. Heart Rhythm..

[15] Linz D., Van Hunnik A., Hohl M., Mahfoud F., Wolf M., Neuberger H.R., Casadei B., Reilly S.N., Verheule S., Böhm M. (2015). Catheter-Based Renal Denervation Reduces Atrial Nerve Sprouting and Complexity of Atrial Fibrillation in Goats. Circ. Arrhythmia Electrophysiol..

[16] Wilson S., Kistler P., Mclellan A.J., Hering D., Schlaich M.P. (2014). Renal Denervation and Pulmonary Vein Isolation in Patients with Drug Resistant Hypertension and Symptomatic Atrial Fibrillation. J. Atr. Fibrillation.

[17] Younis A., Steinberg J.S. (2021). Renal Denervation for Patients with Atrial Fibrillation. Curr. Cardiol. Rep..

[18] Steinberg J.S., Shabanov V., Ponomarev D., Losik D., Ivanickiy E., Kropotkin E., Polyakov K., Ptaszynski P., Keweloh B., Yao C.J. (2020). Effect of Renal Denervation and Catheter Ablation vs. Catheter Ablation Alone on Atrial Fibrillation Recurrence among Patients with Paroxysmal Atrial Fibrillation and Hypertension: The ERADICATE-AF Randomized Clinical Trial. JAMA-J. Am. Med. Assoc..

[19] Yamada S., Lo L.W., Chou Y.H., Lin W.L., Chang S.L., Lin Y.J., Chen S.A. (2017). Renal Denervation Regulates the Atrial Arrhythmogenic Substrates through Reverse Structural Remodeling in Heart Failure Rabbit Model. Int. J. Cardiol..

[20] Mariani M.V., Manzi G., Pierucci N., Laviola D., Piro A., D’Amato A., Filomena D., Matteucci A., Severino P., Miraldi F. (2024). SGLT2i Effect on Atrial Fibrillation: A Network Meta-Analysis of Randomized Controlled Trials. J. Cardiovasc. Electrophysiol..

[21] Mahfoud F., Himmel F., Ukena C., Schunkert H., Böhm M., Weil J. (2011). Treatment Strategies for Resistant Arterial Hypertension. Dtsch. Arztebl. Int..

[22] Ukena C., Becker N., Pavlicek V., Millenaar D., Ewen S., Linz D., Steinberg J.S., Böhm M., Mahfoud F. (2020). Catheter-Based Renal Denervation as Adjunct to Pulmonary Vein Isolation for Treatment of Atrial Fibrillation: A Systematic Review and Meta-Analysis. J. Hypertens..

[23] Romanov A., Pokushalov E., Ponomarev D., Strelnikov A., Shabanov V., Losik D., Karaskov A., Steinberg J.S. (2017). Pulmonary Vein Isolation with Concomitant Renal Artery Denervation Is Associated with Reduction in Both Arterial Blood Pressure and Atrial Fibrillation Burden: Data from Implantable Cardiac Monitor. Cardiovasc. Ther..

[24] Fengler K., Rommel K.P., Blazek S., Besler C., Hartung P., Von Roeder M., Petzold M., Winkler S., Höllriegel R., Desch S. (2019). A Three-Arm Randomized Trial of Different Renal Denervation Devices and Techniques in Patients With Resistant Hypertension (RADIOSOUND-HTN). Circulation.

